# Salivary mental stress biomarkers in COVID-19 patients

**DOI:** 10.3389/fmed.2022.999215

**Published:** 2022-11-02

**Authors:** Tanya Deneva, Youri Ianakiev, Oliana Boykinova

**Affiliations:** ^1^Department of Clinical Laboratory, Medical University of Plovdiv, University Hospital “St. George”, Plovdiv, Bulgaria; ^2^Research Institute at Medical University of Plovdiv, Plovdiv, Bulgaria; ^3^Department of Psychology, University of Plovdiv Paisii Hilendarski, Plovdiv, Bulgaria; ^4^Department of Infection Diseases, Parasitology and Tropical Medicine, Medical University of Plovdiv, University Hospital “St. George”, Plovdiv, Bulgaria

**Keywords:** COVID-19, mental stress, salivary biomarkers, saliva cortisol, saliva alpha-amylase, saliva and chromogranin A

## Abstract

**Background:**

During the COVID-19 pandemic, mental health disorders and level of stress show a major increase compared to before the pandemic. Coronavirus-related stress is recently the leading cause of negative impacts on global mental health. Thus, maintaining positive mental health is as important as maintaining physical health during COVID-19. The aim of this study was to analyze salivary mental stress biomarkers as cortisol, alpha-amylase, and chromogranin A in hospitalized patients with COVID-19 to compare their potential relationship with stress symptoms.

**Material and methods:**

A total of 80 adult hospitalized patients with moderate COVID-19 disease and a control group (*n* = 80) randomly selected were conducted as participants. Saliva cortisol (sCort), saliva alpha-amylase (sAA), and saliva and chromogranin A (sCgA) were determined by the ELISA method (Bio Vendor, USA). Symptoms of stress were measured with a stress symptom checklist (SSCL).

**Results:**

The patients group presented significantly higher levels of sCort, sAA, and sCgA compared with the control group. The correlation analysis showed a positive correlation with strong strength between sCort and sAA (*r* = 0.934, *p* < 0.01), as well as sAA with sCgA (*r* = 0.714, *p* < 0.01). A moderate positive correlation was found between sCort with sCgA (*r* = 0.618, *p* < 0.05). Based on their stress scores from the SSCL the patients were associated with high stress level (30.00%) and very high stress levels (67.5%). In terms of the controls, all the participants showed a low to moderate stress level. We found significant positive correlation between levels of stress and salivary biomarkers.

**Conclusion:**

Data from our study demonstrated that salivary biomarkers are promising tools of exploring COVID-19 related stress.

## Introduction

The stress response triggers a series of psychological, immunological, and biochemical responses that directly affect human health and wellbeing. The COVID-19 pandemic contributes considerably to increasing the number of mental health disorders related to anxiety, depression, distress, and aggressive behavior. Studies show significant levels of stress, anxiety, burnout, fear, and frustration during the pandemic, compared with studies before the pandemic (1–6). Meanwhile, COVID-19 may cause neurological and psychiatric disorders such as stroke, dementia, Parkinson's disease, schizophrenia, cognitive disorders, and bipolar disorders (7). Therefore, people suffering from chronic health diseases are more vulnerable to SARS-CoV-2 infection and they are at higher risk for adverse mental health outcomes (8–10).

Stressful life situations have a negative impact on mental and physical health and lead to serious psychological problems. Such changes in daily life caused by COVID-19 have the potential to increase stress levels with a wide range of psychosocial health problems such as various psychodynamic and physiological dysfunctions (11–14) which occur not only to COVID-19 patients, but also to their family members (15). In this regard, the assessment of the level of stress on the psychological health in patients with coronavirus disease is becoming an essential topic of research in developing effective, reliable, and valid tools for stress assessment.

In recent years, researchers concentrated attention on the evaluation of different proteins in saliva secreted by healthy people and patients with various diseases during response to acute mental stress. Such studies have centered on cortisol, alpha-amylase, chromogranin A, and immunoglobulin A as salivary biomarkers of stress, anxiety, or depressive disorders (16–22). These proteins can be analyzed in other biological specimens, but saliva samples can reflect real time levels of biomarkers with a wide range of their concentration during stress-related disease (23).

Among other salivary stress biomarkers, saliva cortisol (sCort) is most frequently used as a “gold standard marker of stress” (24, 25). The concentration of cortisol in saliva is proportional to the plasma level and it can directly indicate the activity of the hypothalamic-pituitary adrenal axis (HPA) (26).

Alpha-amylase and chromogranin A are other biomarkers representative of activation of the sympathetic-adrenal-medullary (SAM) system which are easily detected in saliva (22, 27). Saliva alpha-amylase (sAA), a digestive enzyme produced and excreted from norepinephrine-responsive salivary gland cells (28), is generally used as a biomarker of psychophysiological stress (27, 29). In recent years, sAA activity has emerged as a valid and reliable marker of sympathetic activation in stress research (28–31).

Chromogranin A (CgA) is the transmembrane glycoprotein belonging to the granin family. It is stored and released with catecholamines into the circulation from secretory vesicles of neurons and endocrine cells upon sympathetic stimulation (32). After being discovered, CgA was initially widely accepted as a biomarker for neuro-endocrine tumors with different primary localization (33, 34). Previous research shows salivary CgA (sCgA) is a sensitive and quantitative index of the activity of the SAM like sA-A (35–37). A variety of studies on stress suggest that elevated levels of sCgA are used as markers of psychological stress (16, 24, 27, 28, 37, 38).

Even though many studies have scientifically proven the potential applicability of sCort, sAA, and sCgA as markers of both psychological and physical stress, data about their use as indicators of activation of the HPA/SAM system during stress response in COVID-19 are still limited. Therefore, more specific reliable studies are needed to assess the stress response not only because of the long-term nature of the virus, but also because of the large scale of the chronic stress associated with COVID-19.

The aim of this study was to analyze salivary mental stress biomarkers as cortisol, chromogranin A, and alpha-amylase in hospitalized patients with COVID-19 and to objectively assess the presence of stress levels using a stress symptom checklist (SSCL) test to compare the potential relationship with salivary biomarkers.

## Materials and methods

### Study design

Our study included 80 adult patients (age ≥ 20 years) with moderate COVID-19 symptoms, who were admitted to our hospital isolation wards between January 2022 and April 2022. The control group of 80 individuals was randomly selected and used as a control to verify the results. All patients with COVID-19 were confirmed by using real-time reverse transcriptase polymerase chain reaction (RT-PCR) assays from oropharyngeal swab specimens. The diagnosis and classification of COVID-19 were based on the Interim Guidance for Clinical Management of COVID-19 issued by the WHO (39). Patients with moderate disease were individuals who showed evidence of lower respiratory disease during clinical assessment or imaging, but no signs of severe pneumonia, including oxygen saturation (SpO_2_) ≥ 90% on room air at sea level. The exclusion criteria for study were primary axis disorders; psychiatric disorders or using psychotropic drugs—anti-depressants, sedatives, or hypnotics; cardiovascular disease, diabetes, cancer, stroke, metabolic, or endocrinological abnormalities.

The control group was composed of volunteers who gave a negative result for COVID-19 by RT–PCR assay and had not suffered from an infection in the last 6 months. All participants were given a document about the objectives and procedures of the study and informed consent was obtained. The study was conducted in accordance with the Declaration of Helsinki and was approved by the local Institutional Ethical Board.

### Instruments

Saliva specimens (Salivette^R^, Sarstedt) were taken in the morning between 6 and 8 AM, within the first 24 h of hospital admission, and second salivary swab was collected on the 10^th^ day of hospitalization. The samples were stored at −20°C until the time of the analysis, but for no longer than 2 months according to the manufacturer's instructions. The manners of withdrawal, processing, and storage of saliva samples were to follow the requirements and the recommendations given by the manufacturer to compensate for the factors of result variation and for standardization of the preanalytical stage.

Salivary stress biomarkers (sCort, sAA, sCgA), were determined using a competitive ELISA assay (BioVendor, USA) after it was validated locally. The methods show high precision; the results are consistent with the recommended minimal non-reproducibility (intra-assay CV < 10%; inter-assay CV < 12%) for ELISA, as given by the manufacturer.

Symptoms of stress were measured with a stress symptom checklist (SSCL) (40, 41). The SSCL is a valid instrument to measure the level of stress based on the number of symptoms that have occurred often enough. Therefore, the patients were examined twice to avoid the possible influence of previous stressful life situations. The SSCL consists of 52 items divided into the following two subscales: physical symptoms (27 items) and psychological symptoms (25 items). Mean values were calculated, and subscales were categorized into “low” (0–7 items checked), “moderate” (8–14 items checked), “high” (15–21 items checked) and “very high” (22+) degrees of stress using the cut-off values suggested by Bourne (40). Higher scores mean a higher level of stress. A total score of 10 on the two sub-scales indicates moderate stress for that person.

### Statistical analysis

Collected data was analyzed using R software, version 4.2. Continuous variables were expressed as median and interquartile ranges (median ± IQR). Qualitative variables were presented as numbers (n) and percentage (%). Column proportions were compared using a two-tailed z-tests. The Shapiro-Wilk test was used to test the normal distribution of all continuous variables. For not-normal distributed variables, we use non-parametric tests (such as Wilcoxon signed rank—for paired observation and Wilcoxon rank sum test). The SSCL was described through the mean value and standard deviation (SD) and as an ordinal variable including the following stress levels: low (0–7), moderate (8–14), high (15–21) and very high (>22). Statistical inference is considered at the level *p* < 0.05.

### Sample size and power analysis

The results of the minimum sample size −67 patients—were calculated to achieve a power of 80% and a level of significance of 5% (two sided), for detecting a mean of 7 points in SSCL differences between pairs, assuming the standard deviation of the differences to be 20. Assumptions are based on literature results validating the stress symptom checklist (SSCL).

## Results

The studied patients group included 44 male (55%) and 36 female (45%) COVID-19 patients. The median age of the patients was 40 years (IQR 36.8–44). The control group consisted of 80 asymptomatic individuals, equally distributed by sex with the median age of 39.5 years (IQR = 37.0, 44.0). No statistical significance between patients' and controls' age, sex, and stress (SSCL) distribution were found. The Wilcoxon Rank Sum Test testing the difference in ranks between patients' and controls' chromogranin A levels, suggests that the effect is positive, statistically significant, and large (*W* = 768, *p* < 0.001; *r* = 0.657). Similar findings also occurred when the test was peformed for amylase (*W* = 861, *p* < 0.001; *r* = 0.632) and cortisol levels (*W* = 1,116, *p* < 0.001; *r* = 0.564) (Table 1).

**Table 1:** Characteristics of the study groups.

**Characteristic**	**Controls,** ***n*** **= 80^a^**	**Patients,** ***n*** **= 80^a^**	**Effect size**	***p*** ^b^
Age	39.5 (37.0, 44.0)	40.0 (36.8, 44.0)	0.008	>0.9
Male	40 (50%)	44 (55%)	0.038	0.5
Cortisol	20.8 (17.1, 22.5)	23.0 (22.4, 25.5)	0.564	<0.001
Chromogranin A	10.9 (7.6, 11.7)	13.7 (13.0, 13.9)	0.657	<0.001
Amylase	12 (9, 15)	23 (18, 29)	0.632	<0.001
Stress	11.50 (8.75, 12.00)	10.00 (8.00, 14.00)	0.048	0.5

a Median (IQR); n (%).

b Wilcoxon Rank Sum test; Pearson's Chi-squared test.

In our study, we used the SSCL to measure the level of stress based on the number of symptoms that have occurred during hospitalization. Out of 25 psychological symptoms included in the SSCL, anxiety was the most common psychological symptom shared by 92.70% of the patients followed by constant worrying by 78.50%, restlessness 69%, frequent irritability 63.40%, and temper flare-ups 61%. Out of 27 physical symptoms in the SSCL, five showed frequencies over 70%: insomnia was reported by 98.50%, backaches by 79.00%, neck pain and tight muscles by 80.00%, muscle cramps by 78.00% and other pain by 72.00%. The remaining 22 physical symptoms were experienced by <50% of the patients and some of them had very low frequency, including: cold feet and hands (3.7%); diarrhea (3.7%); teeth grinding (1.2%); upset stomach (14.6%); and constipation (8.5%).

Based on their stress scores at admission to the hospital there were not significant differences in levels of stress between patients and healthy controls. According to their stress scores on the 10th day of hospitalization −2.5% of the patients were associated with moderate stress levels, 30.00% with high stress levels and 67.5% with very high stress levels. In terms of the controls, all the participants showed a low to moderate stress level (Table 2).

**Table 2:** The extent of the stress level in patients and controls.

**SSCL**	**Patients at admission**	***p***	**Controls**	***p***	**Patients on 10^th^ day**
	**(*n* = 80)**		**(*n* = 80)**		**(*n =* 80)**
**Stress level**					
Low (0–7)	20.0 % (16)	>0.05	22.5% (18)	<0.001	0% (0)
Moderate (8–14)	78,75% (63)	>0.05	77.5% (62)	<0.001	2.5% (2)
High (15–21)	1.25% (1)	>0.05	0% (0)	<0.001	30.0% (24)
Very high (22+)	0 % (0)	>0.05	0% (0)	<0.001	67.5 % (54)

We analyzed the levels of cortisol, chromogranin A and alpha-amylase activity in saliva as potential biomarkers associated with stress during COVID-19. Comparing the patients in the first 24 h at admission and on the 10th day of hospitalization, a statistically significant increase in the levels of the studied substances was found (Table 3). The greatest effect size was found for chromogranin A (0.870, *p* < 0.001) and amylase (ES = 0.870, *p* < 0.001). There was also a significant increase in stress levels measured with the SSCL instrument (ES = 0.869, *p* < 0.001).

**Table 3:** Stress biomarkers in patients.

**Characteristic**	**Patients at admission**,	**Patients on 10^th^ day**,	**Effect size**	***p*** ^b^
	***n*** **= 80^a^**	***n*** **= 80^a^**		
Cortisol	23.0 (22.4, 25.5)	28.9 (25.1, 31.4)	0.753	<0.001
Chromogranin A	13.7 (13.0, 13.9)	20.4 (17.7, 23.8)	0.870	<0.001
Amylase	23 (18, 29)	78 (62, 89)	0.870	<0.001
Stress	10 (8, 14)	25 (20, 30)	0.869	<0.001

a Median (IQR).

b Wilcoxon signed rank test with continuity correction.

**Figure 1:**
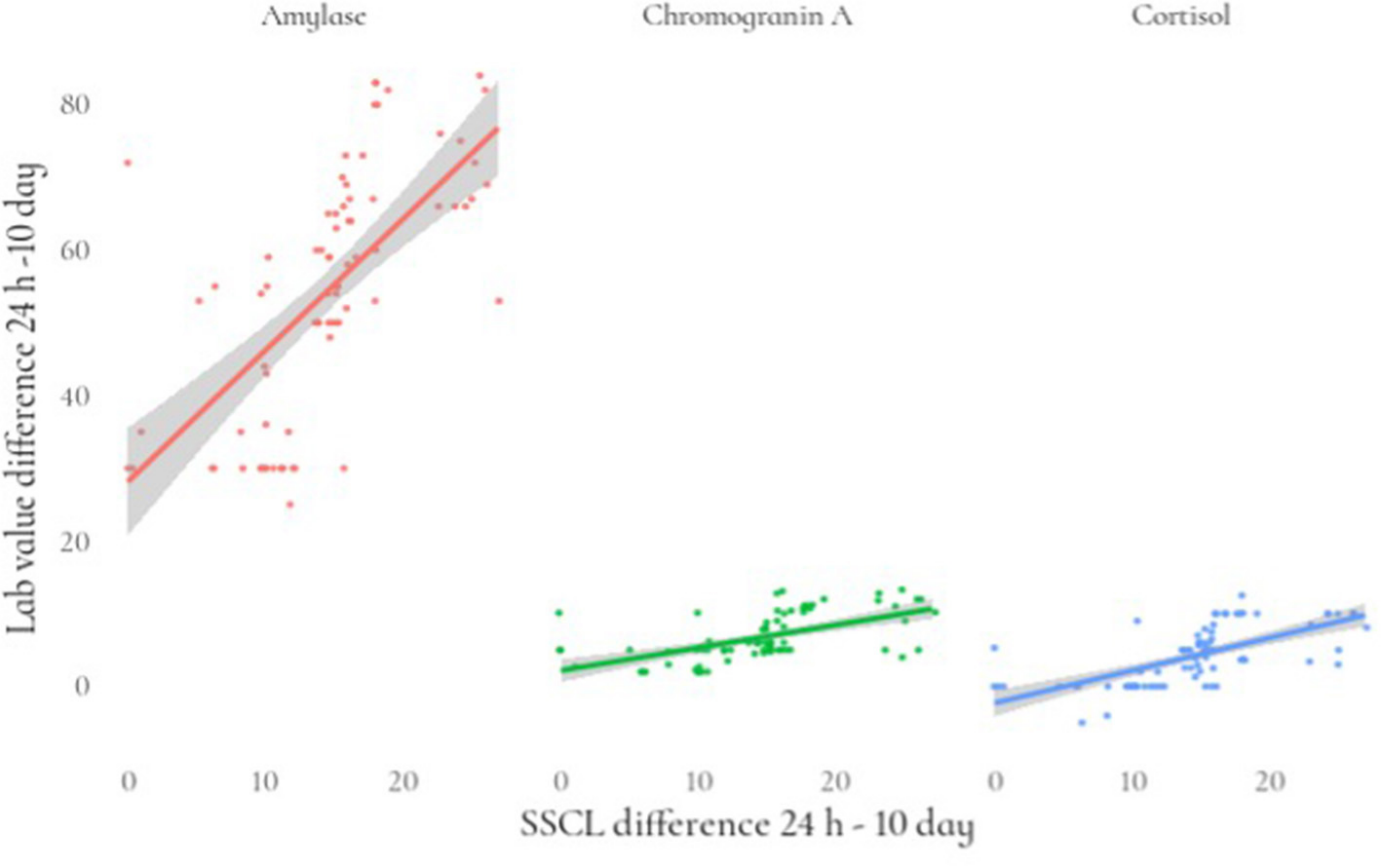
Relationship between the differences (24 h-10 day) in stress levels and lab value of stress biomarkers.

Regarding stress measured by an SSCL, we fitted a linear mixed model (estimated using REML and nloptwrap optimizer) to predict stress with the levels of cortisol, amylase, and chromogranin A. We controlled for sex and age by including them in the model. Standardized parameters were obtained by fitting the model on a standardized version of the dataset, and 95% Confidence Intervals (CIs) and *p* were computed using a Wald t-distribution approximation. The model included patient ID as a random effect in order to account for repeated design. The model's explanatory power related to the fixed effects alone (marginal R^2^) is 0.89. Within this model we estimated the effect of cortisol as statistically significant and positive [beta = 0.39, 95% CI (0.17, 0.61), t_(152)_ = 3.52, *p* < 0.001; Std. beta = 0.17, 95% CI (0.07, 0.26)]. The effect of amylase also was statistically significant and positive [beta = 0.20, 95% CI (0.16, 0.23), t_(152)_ = 10.82, *p* < 0.001; Std. beta = 0.67, 95% CI (0.55, 0.80)] (Table 4; Figure 1).

**Table 4:** Linear mixed model to estimate stress with levels of salivary biomarkers.

**Parameter**	**Coefficient**	**CI_low**	**CI_high**	***t***	***p***	**Std_coefficient**
(Intercept)	−5.699	−11.194	−0.204	−2.049	0.042	−0.043
Age	−0.048	−0.126	0.029	−1.234	0.219	−0.034
Sex [male]	0.667	−0.247	1.581	1.442	0.151	0.078
*Cortisol*	*0.388*	*0.170*	*0.605*	*3.518*	*0.001*	*0.169*
Chromogranin A	0.283	−0.005	0.571	1.942	0.054	0.143
*Amylase*	*0.197*	*0.161*	*0.233*	*10.819*	*0.000*	*0.673*
AIC	821.336					
AICc	822.290					
BIC	845.937					
R2 (marginal)	0.887					
Sigma	2.884					

The values in italic indicate that parameters are significant as opposed to others not in italics.

To determine the relationship between salivary biomarkers in COVID-19 patients, correlation analyses were performed with statistically significant differences between the two groups. A positive correlation with statistical significance was observed between sCort, sAA, and sCgA. The correlation analysis between the stress levels in the SSCL instrument and salivary biomarkers (sCort, sAA, sCgA) showed a significant positive correlation with strong strength between stress levels with sCort and sAA (*r* = 0.961 and *r* = 0.902, *p* < 0.01). A significant positive correlation of moderate strength was shown between levels of stress in SSCL and sCgA (*r* = 0.632, *p* < 0.05) (Table 5). Established correlations also matched in the control group.

**Table 5:** Correlation between biomarkers in patients with COVID-19 (*n* = 80).

**Parameters**	**Salivary cortisol**	**Salivary alpha-amylase**	**Salivary chromogranin A**	**SSCL**
Salivary cortisol	1.000	0.934^**^	0.618^*^	0.961^**^
Salivary alpha-amylase	0.934^**^	1.000	0.714^**^	0.902^**^
Salivary chromogranin A	0.618^*^	0.714^**^	1.000	0.632^*^

** Correlation is significant at the 0.01 level (2-tailed).

* Correlation is significant at the 0.05 level (2-tailed).

## Discussion

The present study on stress and its relationship with COVID-19 infection among hospitalized patients is the first study to examine salivary markers such as sCort, sAA, sCgA together with a psychological stress assessment tool. Our results show that patients with moderate disease have higher levels of salivary biomarkers compared to controls, both at hospital admission and on the 10th day of hospitalization.

Presently salivary cortisol and salivary alpha amylase are scientifically proven as biomarkers of stress and they are used as diagnostic markers not only in acute mental stress, but also in anxiety, depression and PSSD (16, 22, 23, 27). A substantial number of large-scale studies demonstrated that increased level of sCort, sAA, and sCgA indicated the activity of the HPA/SAM system in response to various stress models—psychological, physical, academic, post-traumatic, etc. (17, 28, 38, 42–45). Although sCort and sAA are generally used to indicate activation of the HPA/SAM system, there is more evidence that sCgA is mostly used as a SAM system marker (33, 43, 45).

Despite these studies reporting conflicting responses between sCort, sAA, and sCgA patterns, our results show a significant positive correlation between these three salivary biomarkers, which supports the idea that saliva-based biomarkers could be indicators for dysfunction of the HPA/SAM system during stressor response.

Moreover, in our study to assess potential biomarker variables associated with stress we used an SSCL as a valid, effective instrument measuring non-pathological stress levels in the general population (41). Our results demonstrated that patients have high to very high levels of stress, which are associated with a strong positive correlation with salivary biomarkers. We found no statistical dependence of stress level on age, gender, or diagnosis, despite data reported from global surveys where higher levels of stress were associated with younger age and female gender (9, 46). Rather, these differences may be due not so much to gender and age as to the influence of other environmental factors—socioeconomic, professional and/or family responsibilities, education, and others.

Based on the results of our study, we suggest that the analysis of salivary biomarkers in combination with psychological tools can be useful to achieving a more precise evaluation of the psychophysiological changes and side effects of the stress-induced metabolic abnormalities with increased risk of cardiovascular events, metabolic disease, and neuro-endocrine disease. Therefore, the researchers' attention in the last decade is focused on cardiovascular stress-induced risk produced respectively by the HPA axis and the SAM system. Recent studies propose some evidence that salivary biomarkers were associated with cardiovascular and other metabolic risk factors (47, 48).

Although there is evidence about stress in COVID-19, the extant literature search suffers from studies about the overall biological impact of stress measured by salivary stress biomarkers to examine their association with COVID-19-induced stress. From this perspective, prospective studies and meta-analysis on clinical usefulness of salivary stress biomarkers in COVID-19 should be done to approach key components of the impact of COVID-19 for the mental health.

The results of our study, however, can be interpreted with some limitations. First, it was a single-center, cross-sectional study and therefore it was not possible to analyze the causal relationships between variables. Further, the current study does not take comorbidities into account while estimating salivary stress markers, even though the group of patients was intentionally selected with a moderately severe form, as well as in middle age and history of mental disorders precisely to avoid the influence of comorbidities. Third, we also did not consider the hospital environment influence as an additional source of stress. Moreover, our findings cannot be extrapolated to patients who do not require hospitalization.

Nevertheless, the differences in the levels of salivary biomarkers as well as the correlations found are of high statistical significance compared to the control group and can be used possibly to identify patients with a severe form of disease and comorbidities exposed at risk of chronic stress or psychological problems.

## Conclusion

In hospitalized COVID-19 patients, high levels of mental stress biomarkers sCort, sAA, and sCgA were correlated with high levels of stress symptoms and demonstrated to be a promising tool for good indicators of psychological stress in future stress response assessments during disease outcomes. Further data on the clinical usefulness to making treatment decisions is sparse and needs confirmatory studies.

## Data availability statement

The original contributions presented in the study are included in the article/supplementary material, further inquiries can be directed to the corresponding author.

## Ethics statement

The studies involving human participants were reviewed and approved by Institutional Ethics Committee of Medical University of Plovdiv (Protocol N°1/25.01.2022 and date of approval P-314/02/02/2022). The patients/participants provided their written informed consent to participate in this study.

## Author contributions

TD, YI, and OB designed and coordinated the field study and performed investigation and data curation. TD and YI conceptualized the experiments and performed software, formal analysis, writing, and review and editing. TD designed and performed laboratory assays. TD and OB project administration and funding acquisition. All authors reviewed and approved the final version of the manuscript.

## Funding

This study was supported by Project COV-4/2021—Laboratory monitoring of prognostic biomarkers in hospitalized patients with COVID-19, contract R-2375/02.08.2021, Medical University of Plovdiv and Project COVID-19 HUB—Information, innovations and implementation of integrative research activities, contract KP-06-DK1/6/29.03.2021, National Science Foundation, Ministry of Education and Science.

## Conflict of interest

The authors declare that the research was conducted in the absence of any commercial or financial relationships that could be construed as a potential conflict of interest.

## Publisher's note

All claims expressed in this article are solely those of the authors and do not necessarily represent those of their affiliated organizations, or those of the publisher, the editors and the reviewers. Any product that may be evaluated in this article, or claim that may be made by its manufacturer, is not guaranteed or endorsed by the publisher.
